# “With Every Step, We Grow Stronger”: The Cardiometabolic Benefits of an Indigenous-Led and Community-Based Healthy Lifestyle Intervention

**DOI:** 10.3390/jcm8040422

**Published:** 2019-03-27

**Authors:** Henry P.H. Lai, Rosalin M. Miles, Shannon S.D. Bredin, Kai L. Kaufman, Charlie Z.Y. Chua, Jan Hare, Moss E. Norman, Ryan E. Rhodes, Paul Oh, Darren E.R. Warburton

**Affiliations:** 1 Cardiovascular Physiology and Rehabilitation Laboratory, University of British Columbia, Vancouver, BC V6T 1Z4, Canada; henry.lai@ubc.ca (H.P.H.L.); kai.kaufman@ubc.ca (K.L.K.); charliezongyu@alumni.ubc.ca (C.Z.Y.C.); 2 Indigenous Studies in Kinesiology, Faculty of Education, University of British Columbia, Vancouver, BC V6T 1Z4, Canada; rosalin.miles@ubc.ca (R.M.M.); shannon.bredin@ubc.ca (S.S.D.B.); jan.hare@ubc.ca (J.H.); moss.norman@ubc.ca (M.E.N.); 3 Physical Activity Promotion and Chronic Disease Prevention Unit, Vancouver, BC V6T 1Z4, Canada; 4 Indigenous Physical Activity and Cultural Circle, Vancouver, BC V6N 3S1, Canada; 5 Cognitive and Motor Learning Lab, University of British Columbia, Vancouver, BC V6T 1Z4, Canada; 6 Indigenous Teaching Education Program, University of British Columbia, Vancouver, BC V6T 1Z4, Canada; 7 Behavioural Medicine Lab, University of Victoria, Victoria, BC V8W 3N4, Canada; rhodes@uvic.ca; 8 Cardiovascular Prevention and Rehabilitation Program, University Health Network, Toronto Rehabilitation Institute, Toronto, ON M5G 2A2, Canada; paul.oh@uhn.ca

**Keywords:** community, Indigenous, wellness, cardiorespiratory, cardiometabolic, health, fitness, exercise, behaviour, motivational interviewing, lifestyle counselling

## Abstract

Community-based and Indigenous-led health and wellness approaches have been widely advocated for Indigenous peoples. However, remarkably few Indigenous designed and led interventions exist within the field. The purpose of this study was to evaluate an Indigenous-led and community-based health and wellness intervention in a remote and rural Indigenous community. This protocol was designed by and for Indigenous peoples based on the aspirations of the community (established through sharing circles). A total of 15 participants completed a 13-week walking and healthy lifestyle counselling program (incorporating motivational interviewing) to enhance cardiometabolic health. Measures of moderate-to-vigorous physical activity (MVPA; 7-day accelerometry and self-report), predicted maximal aerobic power (VO_2_max; 6-min walk test), resting heart rate and blood pressure, and other health-related physical fitness measures (musculoskeletal fitness and body composition) were taken before and after the intervention. The intervention led to significant (*p* < 0.05) improvements in VO_2_max (7.1 ± 6.3 % change), with the greatest improvements observed among individuals with lower baseline VO_2_max (*p* < 0.05, *r* = -0.76). Resting heart rate, resting systolic blood pressure, and resting diastolic blood pressure decreased significantly (*p* < 0.05) after the intervention. Self-reported and accelerometry-measured frequency of MVPA increased significantly (*p* < 0.05), and the total MVPA minutes (~275 min/week) were above international recommendations. Change in VO_2_max was significantly correlated with change in self-reported (*r* = 0.42) and accelerometry-measured (*r* = 0.24) MVPA minutes. No significant changes were observed in weight, body mass index, waist circumference, body fat (via bioelectrical impedance), grip strength, and flexibility. These findings demonstrate that a culturally relevant and safe, community-based, Indigenous-led, health and wellness intervention can lead to significant and clinically relevant improvements in cardiometabolic health and physical activity behaviour, with the greatest changes being observed in the least active/fit individuals.

## 1. Introduction

The health benefits of routine physical activity are well established for the general population and those living with chronic medical conditions [1,2,3,4]. As such, regular physical activity participation has been widely advocated for optimal health and wellbeing [5]. Physical activity guidelines [6] have been created for the general population and are often applied with Indigenous peoples. However, recent evidence indicates that generic physical activity guidelines are not culturally relevant or safe for Indigenous peoples, greatly limiting the usage and effectiveness of these guidelines [7].

Over the past decade, individualized exercise prescriptions have been at the forefront of evidence-based best practice in primary and secondary (e.g., clinical exercise rehabilitation) prevention settings. Often, these programs are designed to improve health-related physical fitness, in particular cardiorespiratory fitness [8,9,10,11]. Cardiorespiratory fitness (also referred to as aerobic capacity, maximal aerobic power (VO_2_max), or maximal oxygen consumption) is a strong predictor of cardiometabolic health, premature mortality, and overall quality of life [12,13,14]. Current evidence indicates that cardiorespiratory fitness is declining in Canadian Indigenous populations [15], increasing the risks for several chronic medical conditions and premature mortality. There is growing evidence supporting the need for culturally relevant and safe interventions within Indigenous communities to promote the health-related benefits of routine exercise and/or physical activity participation [16]. Unfortunately, relatively few investigations have been conducted within Indigenous communities that consider the aspirations of the community and/or include Indigenous leaders in the design and implementation of the intervention [16,17]. Moreover, while adhering to individualized exercise prescriptions can lead to marked health-related benefits [2,4], its client-centered approach poses unique cultural challenges in Indigenous community-based programs that use a family-oriented perspective of teaching and learning reflective of local teachings. A family- and community-oriented approach is a cultural, historical, and traditional perspective of teaching and learning valued by diverse Indigenous communities in Canada [18]. As such, culturally safe exercise prescriptions need to adopt a personalized community-based approach because a one-size-fits-all approach to health and wellness should not be applied within Indigenous communities.

Accordingly, the purpose of this study was to evaluate the effectiveness of a community-based and Indigenous designed and led healthy lifestyle intervention on health-related physical fitness within a rural and remote Indigenous community. We hypothesized that participants would exhibit clinically relevant improvements in health-related physical fitness measures, in particular cardiorespiratory fitness. We also hypothesized that the greatest relative changes in health markers would be seen in those with the lowest physical activity and/or fitness levels at baseline.

## 2. Experimental Section

### 2.1. Cocreation of an Indigenous-Led Healthy Lifestyle Intervention

Lytton First Nation is a rural and remote Indigenous community that is located on 14,161 acres divided into 56 reserves that are scattered along a 100 km radius on both sides of the Fraser Canyon [18]. Lytton First Nation is situated in the interior of British Columbia (Canada) northeast of the city of Vancouver. Approximately 270 km of transportation by vehicle was needed to commute to Lytton First Nation from the city of Vancouver.

In consultation and collaboration with Lytton First Nation leaders and community members, we implemented a 13-week walking and healthy lifestyle counselling program including exercise prescriptions designed specifically to improve cardiorespiratory fitness [5]. Unique to this program, our healthy lifestyle intervention was created in partnership with Indigenous Elders and community members as a family-oriented and community-based activity, and implemented in a sharing circle format that is consistent with the Indigenous ways of teaching and learning in Canada [19,20]. Through these sharing circles, our team was able to cocreate a healthy living intervention that built upon the strengths and aspirations of Lytton First Nation community. The healthy lifestyle intervention also made usage of latest advancements in motivational interviewing to enhance the intrinsic motivation to change behaviour such as physical activity participation [21,22,23,24,25]. Integrating the strengths identified by Indigenous communities with current best practices in physical activity promotion, clinical exercise rehabilitation, and motivational interviewing, we cocreated an integrated walking and lifestyle counselling program entitled: “With Every Step, We Grow Stronger.”

### 2.2. Cultural Safety Protocol

Research with Lytton First Nation directly followed the National Aboriginal Health Organization’s health research guidelines related to the principles of ownership, control, access and possession (OCAP^®^) of research data [26]. These principles outline that the research data and knowledge obtained from this study are in the sole possession of Lytton First Nation. All aspects of this study were designed and implemented in consultation and collaboration with Lytton First Nation. All findings were reported back to the community (1/25/19) prior to the submission of this article. These cultural safety and respect protocols responded to 30 of 94 Calls to Action created by the Truth and Reconciliation Commission of Canada to redress the legacy of residential schools and advance the process of Canadian reconciliation with Indigenous communities [27].

### 2.3. Eligibility and Exclusion Criteria

On National Indigenous Peoples Day (6/21/2018), community members from Lytton First Nation (aged 19 years and older) with self-reported Indigenous ancestry (First Nation, Inuit, and Métis) were invited to participate in a 13-week walking and healthy lifestyle counselling program. All participants completed written informed consent and the study was approved by the Clinical Research Ethics board of the University of British Columbia. The study adhered to the guidelines established by the Declaration of Helsinki. Consented participants completed the evidence-based Physical Activity and Readiness Questionnaire for Everyone (PAR-Q+), which is a self-screening tool that is used extensively in Canada and worldwide [28]. On the PAR-Q+, participants answered 7 evidence-based questions regarding their current health status. Answering “No” to all 7 questions indicated that the individual was free of chronic medical conditions, and would receive unrestricted clearance for physical activity participation. Answering “Yes” to 1 or more questions required the completion of additional questions and potential further medical screening by qualified exercise and/or healthcare professionals. A total of 20 healthy adults (free of chronic medical conditions) were self-screened to participate in the program (15 completed the program). All aspects of the program were facilitated by local community leaders, which included Lytton First Nation Band members, restorative justice workers, qualified exercise professionals, and community nurses.

### 2.4. Individualized Exercise Prescriptions

Indigenous community leaders facilitated a group-based walking activity of 30–45 min in duration once per week within the community. Participants were encouraged to invite community members (not part of the intervention) in this family-oriented activity. As such, 15–30 walkers were present in the physical activity component of the intervention. In each walking activity, participants adhered to their individualized exercise prescriptions created by qualified exercise professionals [29]. The qualified exercise professionals received specialized training on cultural respect and safety for work with Indigenous peoples, and provided individualized and group-based consultation regarding exercise safety and progression throughout the intervention. An example 13-week exercise prescription [5] is provided in Table 1. In each individualized prescription, the frequency (3 days/week), intensity (mild-to-moderate), time (15–20 min/day), and type of activity (walking) was the default starting point. The prescription of intensity was based on age, sex, and baseline resting fitness level, the protocol of which is similar to previous exercise prescriptions used with Indigenous peoples [7] and patients with chronic medical conditions (such as heart disease [30] and transplantation [31]). Participants were educated on two methods to monitor exercise intensity: by calculating heart rate reserve (difference between maximum heart rate and resting heart rate) and using the Rating of Perceived Exertion scale (0–10) [32]. Participants were encouraged to meet and exceed the prescribed goals by increasing the frequency (3–5 days/week), intensity (moderate-to-vigorous), duration (30–35 min), and type of exercise (brisk walking) at their own leisure.

### 2.5. Motivational Interviewing and Sharing Circles

Following a group-based walking activity, community leaders facilitated a lifestyle counselling session of 30–45 min in duration to assist participants in setting safe and realistic goals to increase the prescribed frequency, intensity, and duration of walking. A motivational interviewing approach was adopted to enhance the participants’ intrinsic motivation to meet these goals. Motivational interviewing was facilitated in a sharing circle format consistent with the Indigenous ways of teaching and learning [19,20]. Sharing circles are a culturally familiar way of talking in a group setting allowing for the accommodation of Indigenous oral history and storytelling traditions unique to Lytton First Nation [33,34]. Their significance for Indigenous peoples is that talking and listening to one another is the desired interaction. Equally important is that sharing circles are an established cultural practice that create comfort and trust among participants and with the researcher [35]. This method is similar to focus groups in qualitative research, but emphasize participants sharing stories with one another in relation to questions asked to the group [36]. Facilitating motivational interviewing in a sharing circle format allowed for a supportive, empathetic, and directive counselling style that emphasized open-ended questions and reflective listening to help participants discuss their concerns regarding effective healthy lifestyle behaviour change [24,25].

This community-based format enabled participants to share successes and challenges to overcome personal and/or community-based barriers to physical activity participation and healthy living. Through storytelling and knowledge sharing practices, our motivational interviewing approach integrated four of 93 specific behaviour change techniques as identified by the internationally recognized Behaviour Change Technique Taxonomy (BCTT; version 1) [37]: goal setting (e.g., setting goals to meet and exceed the frequency, intensity, duration, and type of exercise prescribed), commitment (e.g., exercise adherence), self-monitoring of behaviour (e.g., self-reporting activity minutes), and self-monitoring of outcome of behaviour (e.g., controlling exercise progression by monitoring exercise intensity). The use of motivational interviewing to increase the health-related benefits of physical activity is consistent with evidence regarding its feasibility to reduce cardiometabolic disease risk in group-based therapy [22].

### 2.6. Cardiorespiratory Fitness and Anthropometry

Cardiorespiratory fitness was evaluated pre- and post-intervention using the submaximal 6-min walk test. During the 6-min walk test, participants were instructed to walk as fast as possible (without breaking into a running stride) repeatedly in linear fashion between a distance of 20 m. Total distance walked was recorded. Total distance and other participant-specific variables were used to compute a predicted VO_2_max value using the following validated equation [38]:

VO_2_max (mL/kg/min) = 70.161 + (0.023 × distance (m)) − (0.276 × weight (kg)) − (6.79 × sex, where m = 0, f = 1) − (0.193 × resting heart rate (bpm)) − (0.191 × age (year)). (1)

On the same day, demographic, anthropometric and health-related physical fitness measures were recorded prior to the 6-min walk test. The third of three measures of resting systolic blood pressure, resting diastolic blood pressure, and resting heart rate were recorded using an automated blood pressure device (BP Tru, Coquitlam, British Columbia, Canada). Weight and body composition (expressed in % body fat; bioelectrical impedance) were recorded using a digital scale (Tanita TBF-300 WA; Tanita, Arlington Heights, IL, USA) that was recalibrated with each measurement. Waist circumference was measured using a standard girth tape by positioning the tape on bare skin at the narrowest point between the bottom of the rib cage and the iliac crest [39]. Grip strength was assessed two times in each hand in alternating fashion using the analog handgrip dynamometer (Almedic, Montreal, Quebec, Canada). Combined maximal left and right grip strength was recorded to the nearest 0.5 kg. Height was measured to the nearest 0.5 cm with the participants standing on bare feet (with feet together and toes pointed outward) on the base of a stadiometer (SECA, Hanover, MD, USA). A sit-and-reach flexibility test of the hamstrings and lower back was performed using a standard flexometer, which required participants to reach forward (via arm extension) and push the sliding marker with the fingertips (measuring to the nearest 0.5 cm) while sitting on the floor with legs extended and bare feet placed flat against the flexometer. Musculoskeletal fitness was assessed using the Canadian Physical Activity, Fitness, and Lifestyle Approach (CPAFLA) fitness norms of Gledhill and Jamnik [40].

### 2.7. Self-Reported and Accelerometry-Measured Physical Activity

The modified Godin-Shephard Leisure Time Activity Questionnaire [41] was used to quantify self-reported (subjective) MVPA minutes pre- and post-intervention. The questionnaire required participants to recall average weekly exercise (frequency and total activity minutes) over the past month. We have used this questionnaire extensively with Indigenous peoples in community-based interventions [15]. Total MVPA minutes were recorded.

The Actigraph wGT3X-BT accelerometer (firmware v1.9.2) (Pensacola, FL, USA) was used to objectively measure the minutes of physical activity intensity in the week pre- and post-program. The accelerometer was worn on the nondominant wrist and was initialized to collect activity counts per minute via a 15s epoch [42]. The accelerometer was worn for seven full days (five weekdays, two weekends), which is the standard wear-time protocol used to measure physical activity intensity [43]. Valid wear-time of less than four days (three weekdays, one weekend) was used as the exclusion criteria [44]. The ActiLife software (v6.13.3) was used to process raw accelerometry data into activity counts using Freedson’s [45] count-based regression model to predict activity intensity for adults. Frequency of sustained MVPA time in bouts of ≥30 min was counted to assess adherence to the prescribed progression of MVPA bouts from 15 to 30 min. Adherence rate was calculated as the number of MVPA bouts ≥ 30 min completed over five bouts as prescribed each week. Total minutes spent in MVPA bouts ≥ 15 min/week (which included bouts of ≥30 min) were used to compare with international recommendations (150 min/week or 30 min/day over five days).

### 2.8. Statistical Analyses

All analyses were performed using SPSS version 24 for Windows (SPSS, Inc., Chicago, IL, USA). A 2 × 3 mixed model ANOVA was used to examine the effects of the intervention on health-related physical fitness (aerobic and musculoskeletal fitness and body composition) and baseline fitness level among three fitness groups. The three fitness groups were created based on normative VO_2_max values established by the American College of Sports Medicine [46]: Moderately Fit (above the 50th percentile of the norm), Unfit (25th to 50th percentile), and Least Fit (below the 25th percentile). Pre- and post-outcome physical activity behaviour measures were compared using paired *t*-tests. The Bonferroni correction test was used in post hoc analyses. For all statistical analyses, an alpha level of *p* < 0.05 was selected a priori. Data are reported as Mean ± Standard Error of the Mean (SEM).

## 3. Results

### 3.1. Cardiorespiratory Fitness and Anthropometric Outcomes

**Figure 1:**
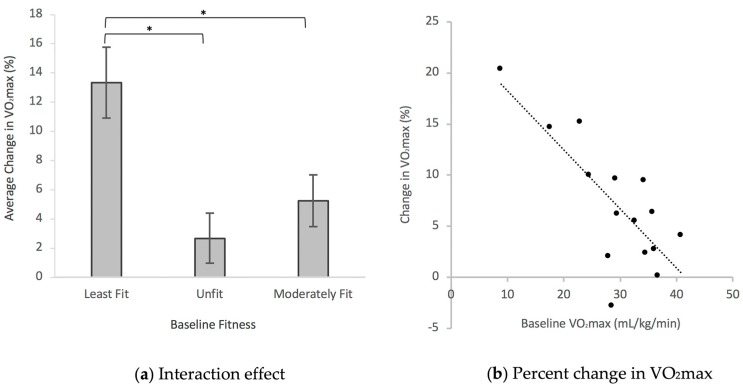
Means and standard error bars are represented, * c*p* < 0.05. (**a**) The greatest improvement in VO_2_max (~13%) was observed in the group with an average baseline VO_2_max below the 25th percentile of the norm. This increase was significantly greater than improvements observed in fitness groups with average baseline VO_2_max values near the 50th percentile of the norm. (**b**) Lower baseline fitness was associated (*p* < 0.05, *r* = −0.76) with greater improvements in VO_2_max.

A total of 15 participants (13 females; *n* = 2, 5, 3, 5 for ages < 30, 30–40, 40–50, >50, respectively) completed the health-related physical fitness and anthropometric tests. There were significant main (time and group) and interaction (time x group) effects for VO_2_max across the intervention (Table 2; Figure 1a). Across all groups, VO_2_max increased (7.1 ± 1.6%) from 29.3 ± 2.1 to 31.0 ± 2.1 mL/kg/min after the 13-week intervention. The least fit group had the lowest VO_2_max at baseline and at follow-up (Table 2). The interaction effect revealed differential responses between groups with the least fit group demonstrating the greatest improvements in VO_2_max (13.3 ± 2.4%) followed by the moderately fit (5.3 ± 1.8%) and unfit (2.7 ± 1.7%) groups. The increase in VO_2_max in the least fit group was significantly greater than the improvements observed in the unfit (*p* = 0.008) and moderately fit (*p* = 0.04) groups. Linear regression analysis demonstrated a significant negative correlation (*p* < 0.05, *r* = −0.76) between change in VO_2_max and baseline values (Figure 1b), supporting the findings that individuals with the lowest cardiorespiratory fitness had the greatest improvements in VO_2_max. There was a significant reduction in resting heart rate, systolic blood pressure, and diastolic blood pressure across the intervention (Table 3).

There were no significant changes in body mass, body mass index, waist circumference, percentage body fat, grip strength, and flexibility following the intervention (Table 4). At baseline, 13 of 15 participants exhibited a BMI > 25 kg/m^2^ (overweight, *n* = 6; obese, *n* = 7), and 12 of 15 participants (12 of 13 females) revealed elevated waist circumference. Among female participants, the baseline average of percentage body fat was 41.7 ± 2.2%, while the baseline percentage body fat of the male participant was 15.2%. Based on the CPAFLA musculoskeletal norms, approximately half the cohort showed a baseline sit-and-reach flexibility rating that was below average (“Needs Improvement”; “Fair”; “Good”; *n* = 7, 4, 4, respectively), while baseline grip strength results were less homogeneous (“Needs Improvement”; “Fair”; “Good”; “Very Good”; “Excellent”; *n* = 5, 3, 1, 4, 2, respectively).

### 3.2. Exercise Adherence and Adverse Events

There were no reported cases of exercise-related adverse events during the 13-week intervention. The average weekly frequency of completing five MVPA bouts of ≥30 min as prescribed increased significantly from 2.4 ± 0.8 to 4.6 ± 1.1 per week (adherence rate of 92.7 ± 21.3%), reflecting a transition from 73.6 ± 24.4 to 139.1 ± 31.9 min/week (Table 5). The MVPA time spent in bouts of ≥30 min increased across the intervention in all groups (Table 5).

### 3.3. Physical Activity Levels Meeting International Recommendations

**Figure 2:**
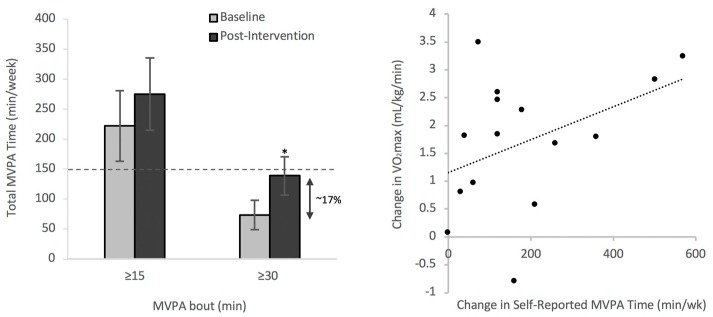
Means and standard error bars are represented, * *p* < 0.05. (**a**) Accelerometry-measured time spent in completing MVPA bouts ≥30 min increased significantly by ~17% (~nine min/day). Total time spent in MVPA bouts ≥ 15 min (including MVPA bouts ≥ 30 min) was above international recommendations of 150 min/week (represented by the dashed line). (**b**) A positive correlation (*p* < 0.05, *r* = 0.42) was observed between change in self-reported MVPA time and change in VO_2_max.

The increase in MVPA minutes completed in bouts of ≥30 min approached international recommendations (Table 5; Figure 2a). There was a main effect for the intervention. There were no interaction effects likely owing (at least in part) to the small sample size for each group. With the inclusion of MVPA minutes completed in bouts of ≥15 min, overall MVPA time (275.5 ± 60.2 min/week) was above international recommendations following the intervention (Table 5). Overall, international physical activity guidelines were met by increasing daily MVPA time (9.4 ± 4.0 min/day; ~17% change) in adherence to exercise bouts ≥30 min (Table 5; Figure 2a).

Significant changes were also observed in self-reported MVPA, in which all fitness groups met international recommendations following the intervention (Table 6). However, there were significant differences between the self-reported and directly assessed (via accelerometry) MVPA (min/week) measures in 11 participants reflecting a marked underestimation via self-report at baseline (−21.7%) and overestimation after the intervention (31.0%). This discrepancy was observed in the number of participants that were above international recommendations of MVPA time (7 of 15 (~46.7%) via self-report and eight of eleven (~72.6%) via accelerometry). Change in VO_2_max was significantly (*p* < 0.05) correlated with change in self-reported (*r* = 0.42; Figure 2b) and accelerometry-measured (*r* = 0.24) MVPA minutes completed in exercise bouts ≥ 30 min. No significant changes were observed in percent change in MVPA and in total Actigraph wear time.

## 4. Discussion

To date, limited studies have directly examined the cardiometabolic benefits of an Indigenous led and community-based healthy lifestyle intervention conducted within a remote and rural Indigenous community. This study is unique in presenting directly measured indices of health-related physical fitness and physical activity behaviour after a 13-week healthy lifestyle intervention. The key findings from this study revealed that a culturally relevant and safe, community-based, Indigenous-led, health and wellness intervention can lead to significant and clinically relevant improvements in cardiometabolic health and physical activity behaviour. Importantly, the greatest benefits occurred in the least fit individuals. The study also showed the potential for this program to prevent against further weight gain. These findings demonstrated that extensive community engagement played a key role in effective behavioural change.

Indigenous-led approaches have been widely advocated to improve cardiorespiratory fitness in Indigenous communities. A recent systematic review of the literature has revealed that the cardiorespiratory fitness of Indigenous peoples may be declining, increasing the risk for chronic disease and premature mortality [14]. In the present study, the increase in aerobic fitness (~7%) would be associated with a clinically relevant reduction in the risk for chronic disease and premature mortality [1,2,3,4,5]. In a position stand articulated by the American College of Sports Medicine [47], a ~7% increase in aerobic fitness is expected following the adherence of exercise prescriptions over a three month period (Table 1). The position stand states that a 10–15% increase in aerobic fitness is typically observed in adherence to 6–12 months of aerobic-based exercise at an intensity of 40–50% HRR (which was the starting point in this study), and that training at this intensity has been shown to elicit significantly greater improvements in aerobic fitness among individuals with lower baseline fitness [47]. Consistent with this trend, we demonstrated that aerobic-based exercise at this starting intensity led to a ~13% increase in aerobic fitness in the least fit group over a three month period. Furthermore, while accelerometry-measured MVPA time doubled in both the least fit and moderately fit groups, the greatest changes in aerobic fitness were observed in the least fit group (Figure 1a). These observations are clinically relevant. For instance, Myers and colleagues revealed risk reductions for premature mortality of 10–25% for every 1-MET increase in aerobic fitness [48], with greater risk reductions (roughly 30% per 1-MET increase) in those with extremely low aerobic fitness levels (e.g., <5 METs). While aerobic fitness is a stronger predictor of the risk for chronic disease and premature mortality than physical activity behaviour [1,2,3,4,5], physical activity behaviour is an important contributor to aerobic fitness.

Various factors explain changes in aerobic fitness with training including physical activity behaviours. In the current study, there were significant increases in both subjectively and objectively measured indices of MVPA. As observed via accelerometry, there was a strong adherence to the exercise prescription by the participants with the entire cohort approaching international recommendations (139.0 ± 31.9 min/week) by completing an average of 4.6 ± 1.1 MVPA bouts of ≥30 min out of the five bouts as prescribed. Overall, the ~17% increase in completing MVPA of ≥30 min demonstrated evidence for health behavioural change in physical activity participation. The ~17% increase in MVPA minutes reflects a ~ten min increase (per day) in the transition from ≥15 to ≥30 min bouts of MVPA as prescribed (Table 5, Figure 2a). A ~ten min daily increase in MVPA has clinical relevance, as international guidelines recommend meeting 30 min/day of MVPA in bouts of ≥10 min to accommodate for individuals with extremely low baseline fitness. While the lack of a control group is a limitation in community-based designs such as this study, a community-based design offers practical, realistic, and replicable methodology to evaluate the impact of adhering to international physical activity guidelines on cardiorespiratory fitness.

Consistent with evidence demonstrating the relationship between minor volumes of MVPA (e.g., threshold of ten min) and significant gains in health-related benefits [1,2,3,4,5], the results in our study revealed that the ~ten min daily increase in MVPA led to predictable changes in health-related physical fitness measures. For instance, the reductions in resting heart rate and blood pressure were similar amongst groups and were within clinically recommended ranges for adults following the intervention (e.g., heart rate = 60–80 bpm and blood pressure less than 120/80 mmHg) [49,50]. Our observations also align with empirical evidence at the highest level. In a recent systematic review of systematic reviews, Warburton and colleagues [2] revealed a dose–response (curvilinear) relationship between physical activity and health status, such that marked health-related benefits were observed with relatively minor volumes (e.g., ten min per day) of physical activity. Research from cardiac rehabilitation settings also suggests that a ten min increase in MVPA can have profound clinical implications [13]. Although changes in MVPA did not explain all of the variance in the changes in aerobic fitness, further research is warranted to fully examine the other mechanisms responsible for the change in aerobic fitness with a healthy lifestyle intervention of this nature.

Another important finding in this study was the misrepresentation of MVPA minutes via self-report. The over-reporting of MVPA minutes is not uncommon [51], as over-estimation of self-reported MVPA by up to 56.8% has been documented [52]. In our study, we observed a marked difference between the subjective and objective measures of MVPA both before and after the intervention. In particular, self-reported MVPA times were underestimated and overestimated pre- and post-intervention, respectively. These observations were distinct from what is frequently observed in the general population (e.g., overestimation in both pre- and post-intervention assessments), and further research is warranted to determine the reasons for underestimation. This finding also emphasizes the need to take objective measures of physical activity when feasible [43].

In addition to significant improvements in cardiorespiratory fitness, this study also showed the potential for this program to prevent against further weight gain. At baseline, the participants demonstrated a body composition that placed them at a higher risk for chronic disease [53] with a graded risk response according to baseline aerobic fitness (e.g., Least Fit > Unfit > Moderately Fit, *p* < 0.05). The observation that health-related benefits could be obtained despite no weight loss is clinically relevant to primary and secondary prevention programs in Indigenous communities. Indigenous peoples have frequently been shown to be at an increased risk for overweight, obesity, and cardiometabolic conditions (such as heart disease and diabetes) [54,55,56]. In British Columbia, our team has previously demonstrated that the prevalence of overweight, obesity, and abdominal obesity was 29.4, 48.6, and 65.1%, respectively in Indigenous men and women [55]. Both obesity and abdominal obesity were significantly greater in rural (e.g., Northern and Interior) regions of British Columbia in comparison to more urban centres. While many research studies focus on the changes in body composition (e.g., weight loss) that occur after an intervention, our findings support the clinical practice of preventing weight gain in the initial stages of a physical activity and/or exercise-training program.

Preventing weight gain in the initial stages of a physical activity intervention is important from two perspectives. Firstly, it is feasible for marked cardiometabolic health benefits to occur with relatively small changes in body composition [4]. In fact, it has been shown that small incremental increases in both volume and intensity of physical activity are associated with larger gains in health-related benefits in previously inactive individuals [1,2,3,4,5,8,13]. Secondly, no weight gain over a four-month period can be considered an important benefit for the attenuation of the risk associated with weight gain. Our present study revealed that the healthy living intervention was sufficient to prevent further weight gain in at-risk participants. This finding is consistent with current weight management recommendations related to the prevention of weight gain. For instance, 45–60 min of MVPA per day are often recommended to prevent weight gain, and 60–90 min of MVPA per day are required to sustain long-term weight loss [8,53]. Dividing the accelerometry-measured MVPA time (~275 min/week) by five (as per international guidelines of 150 min/week over five days) revealed an average of ~55 min/day spent in MVPA, which was within the recommendation to prevent weight gain.

Moreover, increasing evidence has demonstrated a positive relationship between musculoskeletal fitness and health status, particularly markers of disability and functional status [1,5]. As such, we have widely advocated the assessment of musculoskeletal fitness [1,5]. Our current investigation focused on aerobic activities, and as such it is not surprising to find no significant changes in grip strength or flexibility across the intervention. Future research should explore the effectiveness of a combined aerobic and musculoskeletal program in community-based interventions, as it has been shown that the integration of neuromuscular and flexibility exercises can enhance functional status in clinical settings [57]. In particular, musculoskeletal programs should focus on improving and/or maintaining grip strength, since grip strength is positively correlated to performance in activities of daily living [58] and inversely related to premature mortality and developing chronic disability [59,60]. Improving grip strength is also a primary prevention for fall-related injuries (e.g., fragility fractures of the hip and wrist) [61] that can undermine routine physical activity participation especially amongst the elderly. Accordingly, future community-based research should examine the effects of integrating musculoskeletal and flexibility exercises into comprehensive healthy living programs for the primary and secondary prevention of chronic medical conditions.

The findings from this study have important implications for the health and wellbeing of Indigenous peoples. At the community-level, we have demonstrated that using culturally safe and Indigenous led methods to implement exercise prescriptions is effective in eliciting marked improvements in cardiometabolic health (e.g., aerobic fitness, resting heart rate, resting blood pressure). The exercise program was also effective in preventing weight gain. These findings are clinically relevant in primary and secondary prevention programs in Indigenous communities, as they provide novel insight on how to design and implement culturally safe and relevant approaches to harmonize the knowledge translation message of “every little bit of activity counts” [8]. With reference to this key messaging slogan, we cocreated an Indigenous led healthy lifestyle intervention model “With Every Step, We Grow Stronger” to fill knowledge gaps related to healthy lifestyle behaviours (including physical activity) and cultural safety.

Future research should make adaptations that build upon the strengths and weaknesses unique to this study. While a larger sample size will increase statistical power, the retention of program participants may depend on an appropriate ratio of facilitators to participants unique to the community. An increase in the number of participants may require more community leaders to be present to facilitate program activities. Finding community members who are able to participate as program leaders can also be a limitation. These challenges can be overcome via culturally safe community engagement practices [19].

Limitations related to the collection of data were also noted. For instance, motivation and previous performance have been documented as factors that contribute to the variability of distance walked in the 6-min walk test [62]. Furthermore, while the combination of questionnaires and accelerometry provides greater insight on physical activity information [63], compliance in wearing accelerometers can limit the availability of valid data. In this study, 11 of 15 participants wore accelerometers in the pre- and post-program assessments. While control groups may be difficult to establish in community-based research, their inclusion can assist in the comparison of between-group interactions including sex-based differences. However, it is important to highlight that owing to the overwhelming evidence supporting the health benefits of routine physical activity participation, the inclusion of a control group that does not receive these benefits is widely cautioned against.

## 5. Conclusions

Addressing the cardiometabolic health of Indigenous peoples continues to be an important line of enquiry. In our study, integrating exercise prescriptions with healthy lifestyle counselling (via sharing circles) in a community-based program improved cardiorespiratory fitness, and the greatest changes in aerobic fitness were being generally observed in the least fit group. These findings are consistent with the dose–response relationship between physical activity and health status, such that the health-related gains associated with physical activity are greatest among the least fit individuals. Motivational interviewing strategies in lifestyle counselling can be adapted to reflect the culture, traditions, and history unique to the community. Through sharing circles, a motivational interviewing approach can adopt specific behaviour change techniques to empower reflective listening and meaningful dialogue. These established cultural practices are effective in sharing the knowledge, experiences, challenges, and successes that shape healthy lifestyle behaviours in Indigenous communities. Empowering Indigenous leaders to be the key advocates of health behavioural change in their community is a culturally appropriate strategy to advance health and wellness promotion in Indigenous communities.

## 6. Patents

As defined by the National Aboriginal Health Organization’s health research guidelines related to the principles of ownership, control, access and possession (OCAP^®^) of research data [26], all research data and knowledge obtained from this study are in the sole possession of Lytton First Nation.

## Figures and Tables

**Table 1:** Example 13-week exercise prescription.

Program Stage	Week	Frequency (days/week)	Intensity			Duration (min)
			%HRR	RPE	Breathing Rate	
**Initial Stage:** Mild to moderate intensity aerobic exercise	1	3	40–50	3–4	Slightly increased	15–20
	2	3	40–50	3–4	Slightly increased	20–25
	3	3	50–60	3–5	Noticeably increased	20–25
	4	3	50–60	3–5	Noticeably increased	25–30
**Improvement:** Exercise intensity and duration increase with fitness	5–7	4	60–70	3–4	Noticeably increased	25–30
	8–10	4	60–70	3–4	Noticeably increased	30–35
	11–13	3–5	65–75	3–5	Noticeably increased	30–35

HRR: heart rate reserve; RPE: rating of perceived exertion (ten-point scale) [32].

**Table 2:** Change in predicted maximal aerobic power (VO_2_max) based on 6MWT.

Fitness Group	Age (year)	6MWT Distance (m)		VO_2_max (mL/kg/min)		ΔVO_2_max (%)
		Pre	Post	Pre	Post	
Least Fit (*n* = 5)	46.8 ± 5.5	439.0 ± 31.5	470.2 ± 18.1	20.7 ± 3.5	23.1 ± 3.6 **	13.3 ± 2.4 **
Unfit (*n* = 5)	36.2 ± 5.0	569.0 ± 24.1	545.0 ± 24.1	34.9 ± 2.0	35.9 ± 2.4	2.7 ± 1.7
Moderately Fit (*n* = 5)	48.6 ± 7.1	487.4 ± 19.8	496.2 ± 27.3	32.4 ± 1.6	34.1 ± 1.7	5.3 ± 1.8
Overall (*n* = 15)	43.9 ± 3.5	498.5 ± 19.8	503.8 ± 15.0	29.3 ± 2.1	31.0 ± 2.1 *	7.1 ± 1.6 *

6MWT: 6-min walk test; * main effect for intervention *p* < 0.05 (Mean ± SE); ** significant interaction effect.

**Table 3:** Change in resting heart rate and blood pressure.

Fitness Group	Resting Heart Rate (bpm)		Resting SBP (mmHg)		Resting DBP (mmHg)	
	Pre	Post	Pre	Post	Pre	Post
Least Fit (*n* = 5)	84.8 ± 4.3	76.4 ± 5.6 *	135.0 ± 8.3	126.8 ± 5.4	85.6 ± 4.1	76.2 ± 5.9
Unfit (*n* = 5)	79.4 ± 5.7	69.2 ± 3.7 *	127.2 ± 5.7	113.2 ± 3.1	79.8 ± 3.7	74.2 ± 2.3
Moderately Fit (*n* = 5)	77.2 ± 3.8	70.4 ± 2.4 *	110.8 ± 3.3	109.8 ± 2.7	67.8 ± 2.9	63.0 ± 4.2
Overall (*n* = 15)	80.5 ± 2.6	72.0 ± 2.4 *	124.3 ± 4.2	116.6 ± 2.9 *	77.7 ± 2.8	71.1 ± 2.8 *

SBP: systolic blood pressure, DBP: diastolic blood pressure, * *p* < 0.05 (Mean ± SE)

**Table 4:** Change in anthropometric and musculoskeletal outcomes (Mean ± SE).

**Fitness Group**	**Weight** **(kg)**		**Body Mass Index** **(kg/m^2^)**		**Waist Circumference** **(cm)**	
	**Pre**	**Post**	**Pre**	**Post**	**Pre**	**Post**
Least Fit (*n* = 5)	99.5 ± 5.7	99.2 ± 5.7	37.2 ± 2.0	37.1 ± 2.0	119.2 ± 5.2	117.8 ± 3.9
Unfit (*n* = 5)	79.7 ± 4.2	81.2 ± 4.0	28.4 ± 3.0	28.9 ± 3.0	101.0 ± 5.0	103.1 ± 4.7
Moderately Fit (*n* = 5)	65.2 ± 4.2	64.6 ± 4.2	26.2 ± 1.1	25.9 ± 1.2	94.9 ± 3.3	93.0 ± 2.6
Overall (*n* = 15)	81.5 ± 4.5	81.7 ± 4.5	30.6 ± 1.7	30.7 ± 1.7	105.0 ± 3.7	104.6 ± 3.4
**Fitness Group**	**Grip Strength** **(kg)**		**Sit-and-Reach** **(cm)**		**Bioelectrical Impedance** **(%)**	
	**Pre**	**Post**	**Pre**	**Post**	**Pre**	**Post**
Least Fit (*n* = 5)	60.6 ± 7.1	59.8 ± 7.0	24.0 ± 3.1	23.3 ± 3.6	49.2 ± 1.6	49.4 ± 1.4
Unfit (*n* = 5)	71.2 ± 5.9	73.6 ± 3.3	26.1 ± 3.2	27.8 ± 2.5	34.6 ± 6.0	35.7 ± 5.9
Moderately Fit (*n* = 5)	52.4 ± 7.3	47.6 ± 6.5	31.3 ± 1.7	31.0 ± 3.0	34.7 ± 2.4	34.2 ± 2.4
Overall (*n* = 15)	61.4 ± 4.2	60.3 ± 4.2	25.7 ± 1.7	27.1 ± 1.8	39.8 ± 2.7	40.1 ± 2.6

**Table 5:** Change in accelerometry-measured moderate-to-vigorous physical activity (MVPA) frequency and time.

**Fitness Group**	**Frequency of MVPA Bouts ≥ 15 min (#/week)**		**MVPA Time ≥ 15 min (min/week)**		**Change in MVPA Time ≥ 15 min Bouts (min/day)**
	**Pre**	**Post**	**Pre**	**Post**	
Least Fit (*n* = 3)	16.0 ± 11.1	15.7 ± 8.8	240.0 ± 167.0	235.0 ± 132.5	-0.7 ± 5.0
Unfit (*n* = 3)	13.7 ± 4.3	22.0 ± 9.6	205.0 ± 63.8	330.0 ± 144.1	17.9 ± 11.5
Moderately Fit (*n* = 5)	14.8 ± 6.4	17.8 ± 5.9	222.0 ± 95.7	267.0 ± 87.9	6.4 ± 8.6
Overall (*n* = 11)	14.8 ± 3.9	18.4 ± 4.0	222.3 ± 58.5	275.5 ± 60.2	7.6 ± 5.2
**Fitness Group**	**Frequency of MVPA Bouts ≥ 30 min (#/week)**		**MVPA Time ≥ 30 min (min/week)**		**Change in MVPA Time ≥ 30 min Bouts (min/day)**
	**Pre**	**Post**	**Pre**	**Post**	
Least Fit (*n* = 3)	2.3 ± 1.9	5.0 ± 3.1	70.0 ± 55.7	150.0 ± 91.7	11.4 ± 5.2
Unfit (*n* = 3)	3.3 ± 0.3	5.0 ± 2.0	100.0 ± 10.0	150.0 ± 60.0	7.1 ± 7.1
Moderately Fit (*n* = 5)	2.0 ± 1.5	4.2 ± 1.5	60.0 ± 46.5	126.0 ± 43.9	9.4 ± 8.1
Overall (*n* = 11)	2.4 ± 0.8	4.6 ± 1.1 *	73.6 ± 24.4	139.1 ± 31.9 *	9.4 ± 4.0 *

* main effect for intervention *p* < 0.05 (Mean ± SE).

**Table 6:** Change in self-reported MVPA time.

Fitness Group	MVPA Time (min/week)	
	Pre	Post
Least Fit (*n* = 5)	89 ± 17	184 ± 19 *
Unfit (*n* = 5)	316 ± 55	508 ± 70 *
Moderately Fit (*n* = 5)	118 ± 27	392 ± 73
Overall (*n* = 15)	174 ± 52	361 ± 79 *

* *p* < 0.05 (Mean ± SE).
